# 

**Published:** 1891-04-11

**Authors:** 


PRICE ONE PENNY.
No. 237, Vol. X.—April 11th, 1891.
[Registered at the General Post Office as a Newspaper for transmission in the United Kingdom and Abroad.]

WITH EXTRA NURSING SUPPLEMENT.

Per Annum, 6s. 6d.; post free.

Monthly Parts, 6d.; post free 8d.

Ditto per Annum, 8s., post free.

### THE HOSPITAL

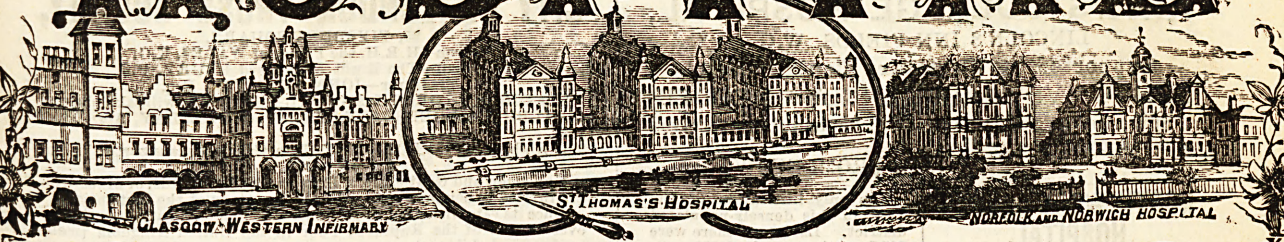

A WEEKLY JOURNAL OF
Science, Medicine, Nursing and Philanthropy.

SATURDAY, APRIL 11th, 1891.

**Shall Children be Insured?** . . . . . . 13

**Passing Topics.**—Hospitals and Lifeboats . . . 14

**The Doctor's Armchair.**—Is Electricity Life? . . 15

**Everybody's Page** . . . . . . . 16

**Poisons and Poisoners,—II.** I.—Arsenic: Madeline Smith (concluded) . . . . . . 17

**Notes and News** . . . . . . . 18

**General Charities—Scraps and Gleanings** . . 19

**Annotations.**—The Abolition of Hypnotism—Science and Lynch Law.—Census Consciences . . . . 20

**The Practitioner's Mirror and Retrospect:—**

MEDICINE.—Epilepsy . . . . . . . 21

SURGERY.—Glottic Spasm—Ingleby Lectures: Lecture I.—Empyema of the Antrum of Highmore . . . 22

NEW DRUGS, APPLIANCES, AND THINGS MEDICAL—Sanitary Rose Powder—Bovinine . . . . . 23

**Radcliffe Infirmary, Oxford** . . . . . . 23

**The Student of Medicine.**—Lectures: Their Use and Abuse—The Students' Bookshelf—Students' Medical Societies—Appointments—Pass Lists.

EXTRA SUPPLEMENT—THE NURSING MIRROR.

**En Passant.**—St. Patrick's Home—Stewardesses—For Prizes or Not?—Nuns versus Nurses—Bravo Bristol!—A Blind Masseuse—A Pleasant Home—The Nurses' Co-operation—Short Items—Paddington Green . . . vii

**Lectures on Surgical Ward Work and Nursing.** By ALEXANDER MILES, M.B.Edin., C.M., F.R.C.S.E. Lecture XX.—Special Cases (continued) . . . viii

**Presentation** . . . . . . . viii

**Death in Our Ranks** . . . . . . viii

**The Eastern Hospital Scandal** . . . . ix

**For Reading to the Sick**—Warning . . . ix

**Male Nurses** (By One of Them) . . . . x

**The Register of Nurses** . . . . . xi

**Notes and Queries** . . . . . . xi

**Buzzing of a "City Bee."** . . . . . xii

**Appointments—Amusements and Relaxation** . . xii

